# Positive perception of aging is a key predictor of quality-of-life in aging people

**DOI:** 10.1371/journal.pone.0204044

**Published:** 2018-10-03

**Authors:** Isabelle Ingrand, Marc Paccalin, Evelyne Liuu, Roger Gil, Pierre Ingrand

**Affiliations:** 1 Pôle Biologie, Pharmacie et Santé Publique, Centre Hospitalier Universitaire de Poitiers, Université de Poitiers, Poitiers, France; 2 INSERM, CIC 1402, Centre Hospitalier Universitaire de Poitiers, Université de Poitiers, Poitiers, France; 3 EA3808 Molecular Targets and Therapeutics of Alzheimer’s disease, Université de Poitiers, Poitiers, France; 4 Pôle de Gériatrie, Centre Hospitalier Universitaire de Poitiers, Université de Poitiers, Poitiers, France; 5 Centre Mémoire de Ressources et de Recherche, Centre Hospitalier Universitaire de Poitiers, Université de Poitiers, Poitiers, France; Iranian Institute for Health Sciences Research, ISLAMIC REPUBLIC OF IRAN

## Abstract

**Objective:**

We conducted a cross-sectional survey in France in a cohort over 55 years of age to characterize the impact of psychological dimensions on quality-of-life (QoL).

**Methods:**

The predictors of QoL in relation with aging were studied using an adapted quality-of-life model, based on emotional, cognitive and physical symptoms, functional status, and general health perception. Adding psychological dimensions such as self-esteem, psychological distress, perceptions of ageing and coping, was hypothesized to improve the QoL model. Responses were analyzed using structural equation modeling and path analysis.

**Results:**

The study involved 258 participants, mean age 66.9±7.9 years. Psychological distress and positive perception of aging exhibited the strongest direct impact on QoL (p<0.0001). Psychological distress also appeared to be mediator on QoL for perceived health status, self-esteem and negative perception of aging. Coping centred on emotion exhibited direct impact on self-esteem and so, indirect impact on QoL (p = 0.0002). Perception of personal financial situation (p = 0.0007) and coping centred on social support (p = 0.02) appeared as direct mediators influencing QoL.

**Conclusions:**

Psychological dimensions are predictors of QOL and have to be taken into account to maximize the resources with a view to successful aging. Further interventions targeting successful aging should focus on positive perception aging.

## Introduction

The number of Europeans aged 65 and over is expected to increase from 17.4% to 25.6% until 2030, and even up to 30% of the population by 2060 due to both the aging of Baby Boomers and increasing life expectancy [1]. Hence, it is central for public health organizations to focus on the quality of life (QoL) of this population. The concept of QoL includes two main dimensions: the feeling of well-being and the health related quality of life (HRQol) which are strong indicators of successful aging [2,3]. The distinction of these two dimensions is interesting since well-being may be more sensitive to psychological aspects than measures of HRQoL. Successful aging does not rely only on clinical health status, but also on psychological and social resources. The importance of individual perceptions of health rather than the sole count of chronic diseases has been shown [4] Determinants of QoL have been studied in the setting of different pathologies using a general model that explores emotional, cognitive, physical symptoms and functional status [5–7]. The integrative analysis of various psychological dimensions such as self-esteem, psychological distress, perceptions of aging and coping, can provide interesting insight into various aspects of life [8–10]. To our knowledge, no previous study yet included the role or the mediation of all of these predictors in the evaluation of the QoL of aging people. Considering their potential impact, it could be useful for practitioners to consider these psychological dimensions to plan appropriate interventions aimed to promote successful aging [4]. The objective of the study was to characterise the relationships of psychological and psychosocial dimensions with the QoL of aging individuals.

## Methods

### Study population and implementation

This survey was proposed to inhabitants aged 55 and over from a community-based population in Poitou-Charentes, France [11]. In the first stage of the study the observatory and its objectives were presented to the medical and social personnel in the area, and then to the population in public meetings. Then, a letter detailing the study was sent to all inhabitants identified from the electoral register, aged 55 or older accompanied by a consent form for participation, or to signal the desire not to participate. In case of agreement to participate but difficulty in reading or writing responses, a third party could assist. After informed consent, anonymous questionnaires were consecutively mailed to participants willing to participate. Questionnaires collected their socio-demographic characteristics, their perceptions of aging and perceived health, quality-of-life, well-being, self-esteem and perceptions of their own socio-economic status. Questionnaires were sent in 3 different mailings during a 12 months period.

An interview aiming to describe cognitive performances, medical context, coping strategies and psychological distress was administered by a psychologist and lasted around one hour. Participants in nursing homes and those for whom the interview evidenced cognitive impairment (notably Mini Mental State Examination score under 24) were not included in the study. The study was approved by the French regulatory authorities on medical research and personal data, the *Comité Consultatif sur le Traitement de l’Information en matière de Recherche dans le domaine de la Santé* (CCTIRS) then the *Commission Nationale Informatique et Liberté* (CNIL).

### Global QoL

Quality-of-Life was measured using the CASP-12 quality-of-life scale which has been validated in aged populations [12,13]. Subjective well-being was studied using the LSI-A (Life Satisfaction Index A), an 11-item scale presenting satisfactory psychometric characteristics [14].

### Determinants of QoL

**Psychological distress** was evaluated by the Hospital Anxiety and Depression Scale (HAD) [15].

**Perceived health** was measured on a visual analogue scale (EuroQol scale) graduated from 0 (worst possible imaginable health state) to 100 (best possible imaginable health state).

**The family support network** (e*nvironmental characteristics*) was rated according to the number of contacts with the family: at least one contact per week; less than one contact per week; less than one contact per month. This scale was derived from the social network index elaborated in the context of the Gazel cohort by Melchior et al [16].

**Financial status** was evaluated *via* the person's subjective perception (fairly well-off, or income adequate to meet fundamental needs, or difficult financial situation).

**Coping**: The Brief COPE, was designed to identify the coping strategies that individuals use the most often. This questionnaire enables the assessment of different forms of coping: particularly coping centred on seeking social support, coping centred on the problem, and coping centred on emotion which aims to reduce or regulate the emotional reaction to the situation [17].

**Self-esteem**, which is an important aspect in facing up to aging 9 was approached using Rosenberg's Self-Esteem Scale (RSES) which enables evaluation of how far the subject generally views him/herself as a person of value [18].

**Perception of aging** was assessed using the Ageing Perceptions Questionnaire (APQ) [11,19]. The APQ has 7 dimensions:

- Timelineness: individual’s awareness of aging, with two sub-dimensions: timeline chronic (awareness of one’s age) and timeline cyclical (experiences of variations in the awareness of aging).
- Consequences: beliefs about the impact of aging on one’s life with two sub-dimensions: positive and negative consequences.
- Control: beliefs about personal ways of managing experience of aging, with two sub-dimensions: positive and negative control.
- Emotional representations: emotional responses generated by aging.

### Statistical analysis

The variables relating to socio-demographic, socio-economic and psycho-social characteristics were described by way of means and standard deviation for quantitative variables, and by numbers and percentages for class variables. At first a bivariate analysis was conducted to describe the correlations between variables relating to perceptions (Brief COPE, RSES, APQ, HAD) and global QoL (CASP-12 and LSI-A).

The structural equation modeling (SEM) approach is used to assess unobservable (latent) variables that are estimated from related observed (manifest) variables. This kind of model is appropriate to investigate the nature of the relationships between QoL and its predictors. A SEM model involves two levels of relationships: the first level (the measurement model) takes into account the relationships between the manifest variables and the corresponding latent variables, the second level (the structural model) considers the relationships among the latent variables [20].

The manifest variables were the validated dimensions of ageing perceptions, coping, psychological distress and quality-of life. The construction of latent variables from these manifest variables was assessed by factor analysis. The structural equations representing hypothesized relationships are tested using a t-statistic computed from the standardized coefficients of path-effect estimates and their standard errors.

To assess the global fit of the model, three criteria were used: i) the goodness of fit χ^2^ test which is based on the discrepancy between the observed model and a model with perfect fit, ii) the root mean square error approximation (RMSEA), and iii) the Bentler comparative fit index (BCFI) [21]. The statistical analyses were performed on SAS software version 9.3 for Windows.

## Results

Among 503 individuals requested to participate in the study, 351 (70%) agreed to take part and 258 of them (74%) fully completed the questionnaires.

### Population characteristics

The mean age of the respondents was 66.9±7.9 years (Table 1). Perceived health scores were generally good (75.0±16.1). QoL, based on the CASP-12 total score, exhibited a mean value of 38.3±5.0 (73% of the maximum score). The mean score for subjective well-being (LSI-A) was 29.7±6.9 (54% of the maximum score).

**Table 1:** Socio-demographic, socioeconomic and psychosocial characteristics of respondents (N = 258).

		n	%
**Socio-demographic characteristics**			
Gender	Male	137	53.1%
	Female	121	46.9%
Diploma (n = 251/258)	< baccalauréat	167	66.5%
	≥ baccalauréat	84	33.5%
Marital status	Living with partner	205	79.5%
	Living alone	53	20.5%
Age (yrs)	Mean(sd)	66.9 (7.9)	
**Family support network** (n = 253/258)			
at least one contact per week with a family member		213	84.2%
less than one contact per week		34	13.4%
less than one contact per month		6	2.4%
**Perception of financial situation**			
Fairly well-off		43	16.7%
Income sufficient for self/family		186	72.1%
Experiencing difficulties		29	11.2%
		Mean	SD
**Health-related QoL (CASP-12) / 48**		38.3	5.0
**Subjective well-being (LSI-TA) / 55**		29.7	6.9
**Perception health status (EQ) / 100**		75.0	16.1
**Self-esteem (Rosenberg scale)/ 40**		30.8	4.5
**Psychological distress (HAD)**			
Anxiety		4.77	3.88
Depression		2.19	3.08
**Aging Perception (APQ)**			
Timeline chronic / 5		2.58	0.83
Timeline cyclical / 5		2.51	0.99
Emotional representations / 5		2.26	0.88
Control negative / 5		3.43	0.69
Consequence negative / 5		2.71	0.96
Control positive / 5		4.10	0.57
Consequence positive / 5		3.36	0.77
**Coping (Brief Cope)**			
Seeking emotional social support / 8		4.29	1.64
Seeking instrumental social support / 8		4.74	1.74
Venting feelings / 8		4.06	1.82
Active Coping / 8		6.68	1.19
Planning / 8		5.50	1.83
Self-Distraction / 8		4.28	1.90
Positive reframing/ 8		6.21	1.53
Humour / 8		5.22	1.26
Acceptation / 8		7.37	0.95
Denial / 8		2.39	0.80
Substance use / 8		2.11	0.49
Disengagement / 8		2.27	0.70
Blame / 8		3.83	1.15
Religion / 8		2.96	1.62

### Determinants of QoL

Bivariate analysis evidenced significant associations between perceptions of health, self-esteem, psychological distress, ageing perception and global QoL (CASP-12 and LSI-A) (Table 2). The dimensions seeking social emotional support and venting feelings in the COPE scale were also related with global QoL.

**Table 2:** Correlation between perception of health status, self esteem, psychological distress, ageing perception, coping and global QoL (N = 258).

	Quality of life	Subjective well-being
	r_spearman_, p	r_spearman_, p
**Perception health status**	**r**_**s**_ **= 0.48 p<0.0001**	**r**_**s**_ **= 0.41 p<0.0001**
**Self-esteem**	**r**_**s**_ **= 0.39 p<0.0001**	**r**_**s**_ **= 0.43 p<0.0001**
**Psychological distress**		
Anxiety	**r**_**s**_ **= -0.17 p = 0.0049**	**r**_**s**_ **= -0.21 p = 0.0007**
Depression	**r**_**s**_ **= -0.36 p<0.0001**	**r**_**s**_ **= -0.37 p<0.0001**
**Aging perception**		
Timeline chronic	**r**_**s**_ **= -0.23 p = 0.0001**	**r**_**s**_ **= -0.27 p<0.0001**
Timeline cyclical	**r**_**s**_ **= -0.23 p = 0.0002**	**r**_**s**_ **= -0.32 p<0.0001**
Representation emotional	**r**_**s**_ **= -0.18 p = 0.0034**	**r**_**s**_ **= -0.33 p<0.0001**
Control negative	**r**_**s**_ **= 0.23 p = 0.0001**	**r**_**s**_ **= 0.28 p<0.0001**
Consequence positive	**r**_**s**_ **= 0.19 p = 0.0025**	**r**_**s**_ **= 0.14 p = 0.030**
Control positive	**r**_**s**_ **= 0.22 p = 0.0003**	**r**_**s**_ **= 0.26 p<0.0001**
Consequence negative	**r**_**s**_ **= -0.37 p<0.0001**	**r**_**s**_ **= -0.32 p<0.0001**
**Coping**		
Seeking emotional social support	**r**_**s**_ **= 0.14 p = 0.022**	**r**_**s**_ **= 0.14 p = 0.028**
Seeking instrumental social support	r_s_ = 0.083 p = 0.18	r_s_ = 0.066 p = 0.29
Venting feelings	**r**_**s**_ **= 0.22 p = 0.0003**	**r**_**s**_ **= 0.13 p = 0.042**
Active Coping	r_s_ = -0.056 p = 0.37	r_s_ = -0.078 p = 0.21
Planning	r_s_ = -0.028 p = 0.96	r_s_ = -0.024 p = 0.70
Self-distraction	r_s_ = -0.029 p = 0.64	r_s_ = -0.018 p = 0.77
Positive reframing	r_s_ = -0.090 p = 0.15	r_s_ = 0.029 p = 0.64
Humour	r_s_ = 0.028 p = 0.66	r_s_ = 0.032 p = 0.61
Acceptation	r_s_ = 0.099 p = 0.11	**r**_**s**_ **= 0.14 p = 0.020**
Denial	**r**_**s**_ **= -0.15 p = 0.013**	r_s_ = -0.091 p = 0.15
Substance use	r_s_ = 0.087 p = 0.16	r_s_ = 0.082 p = 0.19
Disengagement	r_s_ = -0.069 p = 0.27	r_s_ = -0.0029 p = 0.96
Blame	r_s_ = 0.11 p = 0.080	r_s_ = -0.012 p = 0.85
Religion	r_s_ = -0.036 p = 0.57	r_s_ = -0.025 p = 0.69

### Structural equation modeling: Predictors of QoL

From the dimensions of the perception of aging and coping respectively 2 and 3 latent variables were generated (Table 3). These variables were positive and negative representations of aging, coping centred on the social support, on the problem and on emotion.

**Table 3:** Construction of latent variables (factor analysis) from the dimensions observed: Ageing perception, coping and global QoL (n = 258).

	Negative representation	Positive representation	
**Ageing perception**			
Negative representation of ageing			
Timeline chronic	0.72		
Timeline cyclical	0.79		
Representation emotional	0.75		
Control negative	-0.69		
Consequence negative	0.77		
Positive representation of ageing			
Control positive		0.76	
Consequence positive		0.79	
	Coping centred on social support	Coping centred on the problem	Coping centred on the emotions
Coping*			
Seeking emotional social support	0.88		
Seeking instrumental social support	0.81		
Venting feelings	0.82		
Active coping		0.94	
Planning		0.93	
Self-distraction			0.30
Positive reframing			0.87
Humour			0.88
Acceptation			0.48

* the dimensions denial, substance use, disengagement, blame and religion, corresponding to the least frequently used coping strategies (low scores <4), and isolated on the three other factors with loadings under 0.30, were not retained in the multivariate analysis.

The first step in SEM analysis was to verify the relationships between manifest variables and latent variables (measurement model, Table 4). Five latent variables had significant path estimates with t-values greater than 1.96: negative and positive representations of ageing, coping centred on social support, and on emotion, and psychological distress. The coping centred on the problem was not significant.

**Table 4:** Relationships between manifest variables (observable indicators) and latent variables (constructs) (measurement model).

	Negative representation of ageing	Positive representation of ageing	Coping centred on social support	Coping centred on the emotions	Psychological distress	Global QoL
path effect estimate (t value) *						
Timeline chronic	0.67 (15.0)					
Timeline cyclical	0.62 (9.9)					
Emotional representation	0.49 (8.7)					
Control negative	-0.64 (10.6)					
Consequence negative	0.78 (20.6)					
Control positive		0.48 (5.9)				
Consequence positive		0.36 (4.6)				
Seeking emotional social support			0.86 (24.5)			
Seeking instrumental social support			0.67 (15.8)			
Venting feelings			0.76 (20.0)			
Self-Distraction				0.27 (3.2)		
Positive Reframing				0.58 (5.5)		
Humour				0.54 (5.2)		
Acceptation				0.33 (3.8)		
HAD-Anxiety					0.45 (8.2)	
HAD-Depression					0.81 (8.2)	
QoL (CASP-12)						0.73 (16.0)
Subjective well-being						0.78 (19.6)

*To validate the relationships, the t values must be examined for statistical significance. Using normal approximation, t values with their values higher than 1.96 are significantly different from zero. All path estimates are significant (t>1.96).

**Fig 1:**
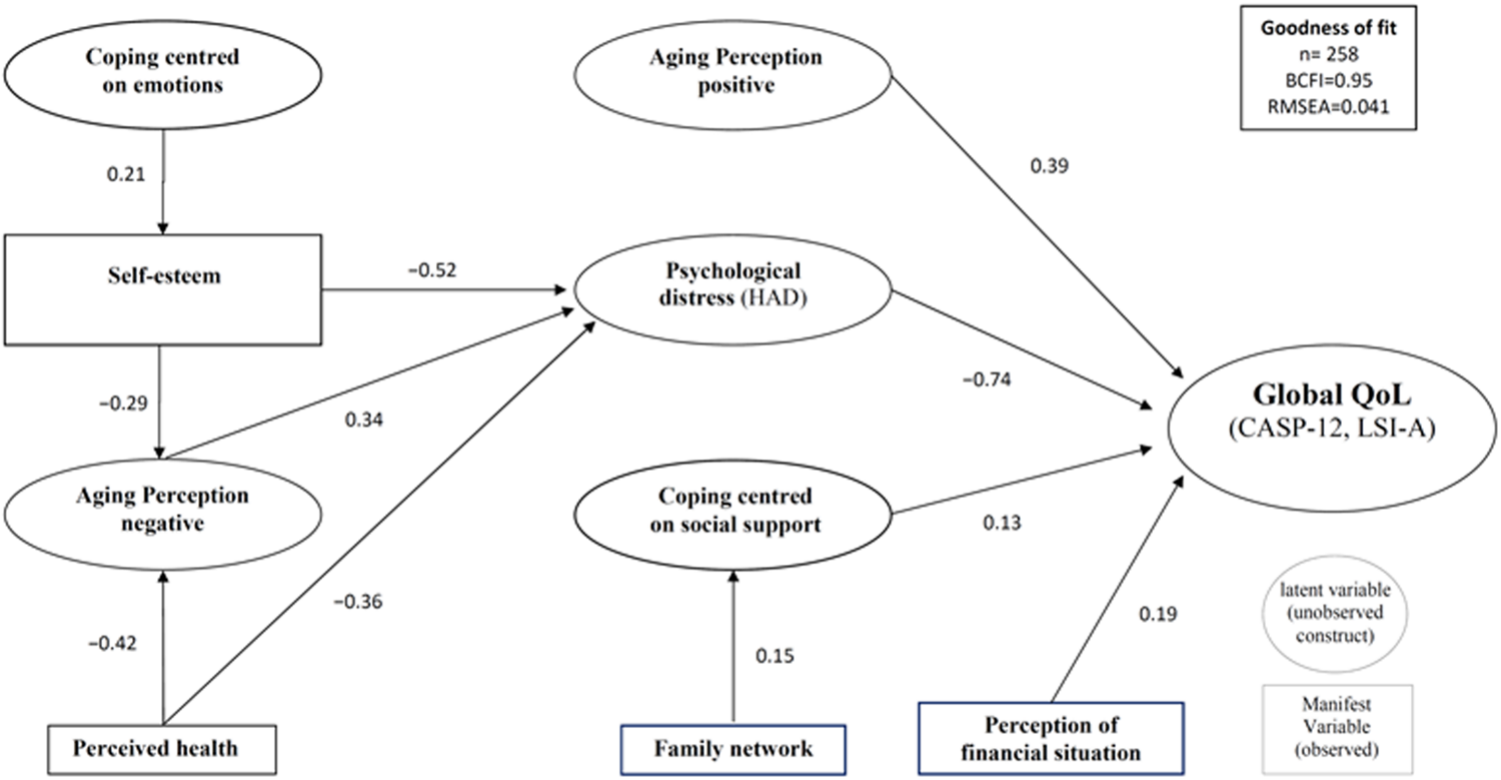
Structural model of predictors of quality of life in aging people. Reported values are parameter estimates of the effects model. All path estimates are significant (t>1.96). Indirect and total effects are not reported on the graph. Fit indices for RMSEA (under 0.05) and Bentler’s CFI (above 0.90) indicate a well fitted model.

The second step in the analysis consisted in validating relationships among latent variables (structural model, Fig 1). In this model, the previous latent variables and the manifest variables (self-esteem, perceived health, family support network and perception of financial situation) were the predictors of global QoL. The final model, including covariance estimates, fitted well the data (RMSEA < .05; BCFI > .90), opening the way to a valid interpretation. As displayed on Fig 1, QoL was related to psychological distress, perceptions of aging, coping and self-esteem. Psychological distress (p < .0001) and positive perceptions of aging (p < .0001) exhibited the strongest direct effect with global quality-of-life in this aging population. Perception of personal financial situation (p = .0007) and coping centred on seeking social support (p = .020) also showed a direct effect. Self-esteem and negative perceptions of aging had significant direct effects on psychological distress (p < .0001 and p = .0001) and significant indirect effects on global quality-of-life (p < .0001 and p = .0002). Coping centred on emotion exhibited direct effects on self-esteem (p = .0009) and indirect effects on global quality-of-life (p = .0002).

## Discussion

The purpose of this study was to analyze how psychological and social factors, not always considered in the clinical model of health, influence QoL in aging people. Psychological distress and positive perception of aging exhibited the strongest direct impact on QoL. Psychological distress also appeared to be the mediator for perceived health status, self-esteem and negative perception of aging on QoL.

This study confirms the impact of psychological distress as a major negative predictor of QoL [22,23]. The main result of our study is the direct significant effect of positive aging perception on QoL without any mediation through other psychological dimensions. Positive perceptions of aging are challenged by the pervasive negative stereotypes of old age in society [24]. It was also shown that individuals with a positive perception of their own aging were less vulnerable to the activation of a negative older adult stereotype in the stereotype threat condition [25]. In community-dwelling older people (65+) in Ireland, higher QoL scores were associated with more positive perceptions of aging [26]. This could well reflect that the individual exerts control over his/her choices which has a positive effect on emotional well-being and the importance for respondents of being self-confident [27,28]. Aging perceptions were shown to affect physical morbidity and mortality. The Ohio Longitudinal Study of Aging and Retirement, a community-based study of individuals aged 50 and older, suggested that self perception of aging predicts functional health over time and that the median survival of individuals with a positive perception of aging was 7.6 years longer than for the ones with negative self perception [29,30].

Other dimensions as perceived health status, self-esteem and negative perception of aging, appeared to be mediated by psychological distress. Self-esteem is indeed strongly, positively linked to subjective well-being [10,31], and negatively correlated with anxiety and depression, with the feeling of poor personal worth [10]. Our results also highlight the role of coping centred on emotion, rather than coping centred on the problem. Emotional coping strategies, calling on positive personal strategies (self-distraction, positive reframing, acceptation, humor) had a significant effect on self-esteem. This could be explained by the fact that aging people use less active strategies, are less focused on problem-solving or seeking assistance, and tend to resort to more passive intrapersonal coping strategies [32]. Perceptions of one's personal financial situation as well as of the family support network and coping centred on social support also have impact on QoL, as already shown [13,22,33–36].

Previous studies have dealt with well being and QoL, but our study resulted in a more comprehensive model, taking into account more psychological dimensions that directly or indirectly mediated QoL [37–41]. Several limits have to be taken into consideration. The selected study population, only included participants without cognitive impairment and 26% of questionnaires were not fully fulfilled. The French socio-cultural context may have considerable differences with other countries, as the results of studies are highly dependent on the cultural perception of aging [22,42,43]. Cross-cultural validation should be required, although similar mediated relationships have been found across 20 countries [44].

It is important to consider how these results may influence programs to support successful aging. Our results could provide an integrative framework for the design of preventive actions aimed to promote successful aging. Positive aging perception has a direct significant effect on QoL without any mediation through other psychological dimensions, including the psychological distress. The results of a previous study provided support for interventions aimed at improving self-perceptions related to efficacy and aging in order to reduce depressive symptoms in older adults [45].

Our results may suggest how to act positively in order to enhance QoL, after an assessment on situations that occur as a person grows old (psychological and environmental status), via i) improvement in the perceptions of aging, ii) bolstering of self-esteem, using coping strategies centred on the problem, and iii) a strengthening of the supporting environment. Future research is required to confirm the predictive value of these determinants in longitudinal surveys and to evaluate their benefit in interventional studies. Although numerous events can occur in a lifetime, including the later years, it is never too late to plan action to improve those aspects contributing to QoL [34]. Successful aging could be bolstered by suggesting programs to build skills in these areas [36]. However conflicting results have been reported and a recent study showed that aging self-perception is not easily manipulated by stereotypes priming. The earlier the actions are implemented the greater the impact, since the characteristics of natural aging are modifiable [46]. This is why we chose to focus on participants aged 55 and over, giving a relatively young mean age (66.9 yrs).

## Conclusion

A comprehensive approach of psychological dimensions related to QoL in aging people enabled to propose an original model. These results may have considerable implications in accompanying aging individuals to maximize their psychological resources with a view to successful aging. The perception of aging, psychological distress, self-esteem, and coping strategies are elements to be taken into account in this perspective.

## Supporting information

S1 Table All data collected are within the supporting information file (supporting data.xls). Gender (female, Male); Perceived health status measured on a visual analogue scale (EuroQol scale); Dimensions of the Ageing Perceptions Questionnaire [19]: Timelineness with two sub-dimensions: timeline chronic and timeline cyclical, emotional representations, positive and negative control, positive and negative consequences; Subjective well-being with the Life Satisfaction Index A [14]; Self Esteem with the Rosenberg's Self-Esteem Scale [18]; Health related quality of life measured using the CASP-12 quality-of-life scale [12,13]; The family support network was rated according to the number of contacts with the family; 3: at least one contact per week; 2: less than one contact per week; 1: less than one contact per month [16]; Psychological distress evaluated by the Hospital Anxiety and Depression Scale [15]; Coping dimensions [17]: self-distraction, active coping, emotional social support, instrumental social support, venting feelings, positive reframing, planning, humour, acceptation.
(XLS)
